# Parallelized volumetric fluorescence microscopy with a reconfigurable coded incoherent light-sheet array

**DOI:** 10.1038/s41377-020-0245-8

**Published:** 2020-01-20

**Authors:** Yu-Xuan Ren, Jianglai Wu, Queenie T. K. Lai, Hei Ming Lai, Dickson M. D. Siu, Wutian Wu, Kenneth K. Y. Wong, Kevin K. Tsia

**Affiliations:** 1 Department of Electrical and Electronic Engineering, The University of Hong Kong, Pokfulam Road, Hong Kong, SAR 999077 China; 2 Department of Physics, University of California, Berkeley, CA 94720 USA; 3 School of Biomedical Sciences, Li Ka Shing Faculty of Medicine, The University of Hong Kong, Hong Kong, SAR 999077 China; 4 Department of Psychiatry, Faculty of Medicine, The Chinese University of Hong Kong, Hong Kong, SAR 999077 China; 5 GHM Institute of CNS Regeneration, Jinan University, 601 Huangpu Avenue West, Guangzhou, 510632 China; 6 Re-Stem Biotechnology, Suzhou, 215007 China

**Keywords:** Biophotonics, Light-sheet microscopy

## Abstract

Parallelized fluorescence imaging has been a long-standing pursuit that can address the unmet need for a comprehensive three-dimensional (3D) visualization of dynamical biological processes with minimal photodamage. However, the available approaches are limited to incomplete parallelization in only two dimensions or sparse sampling in three dimensions. We hereby develop a novel fluorescence imaging approach, called coded light-sheet array microscopy (CLAM), which allows complete parallelized 3D imaging without mechanical scanning. Harnessing the concept of an “infinity mirror”, CLAM generates a light-sheet array with controllable sheet density and degree of coherence. Thus, CLAM circumvents the common complications of multiple coherent light-sheet generation in terms of dedicated wavefront engineering and mechanical dithering/scanning. Moreover, the encoding of multiplexed optical sections in CLAM allows the synchronous capture of all sectioned images within the imaged volume. We demonstrate the utility of CLAM in different imaging scenarios, including a light-scattering medium, an optically cleared tissue, and microparticles in fluidic flow. CLAM can maximize the signal-to-noise ratio and the spatial duty cycle, and also provides a further reduction in photobleaching compared to the major scanning-based 3D imaging systems. The flexible implementation of CLAM regarding both hardware and software ensures compatibility with any light-sheet imaging modality and could thus be instrumental in a multitude of areas in biological research.

## Microscopy: parallel light sheets give a gentler view

A new imaging technique called coded light-sheet array microscopy (CLAM) brings an improved ability to visualize biological samples in three dimensions while minimizing the damage. A research team, led by Kevin Tsia at the University of Hong Kong, developed the technique and demonstrated its use on a variety of biological imaging scenarios. CLAM is a new form of 3D fluorescence microscopy, which collects all the fluorescence signals from the entire illuminated volume of a sample in parallel. Its key innovation is the way it uses a concept called “infinity mirror” to generate an array of parallel slices of laser light (“light sheets”) for three-dimensional imaging without the need for repetitive scanning and with much gentler illumination than the state-of-the-art techniques. CLAM could be applied to existing light sheet fluorescence microscopes, with minimal system modifications.

## Introduction

The grand challenge of understanding the animate processes of complex biological systems in three dimensions lies in the lack of imaging modalities that monitor the dynamics at a sufficiently high spatiotemporal resolution and with a low level of photodamage. Established three-dimensional (3D) biological imaging techniques, namely, confocal and multiphoton microscopy and light-sheet fluorescence microscopy (LSFM)^1–3^, predominantly rely on laser scanning that often compromises the imaging speed because the entire 3D field of view (FOV) has to be sequentially scanned by mechanical motions involving a galvanometric scanner or a bulky imaging lens. While assorted techniques have been developed to scale the scanning speed^4–6^, the techniques inevitably require higher system complexity, including dedicated beam-scanning control and hardware synchronization. Indeed, laser-scanning imaging is intrinsically power-inefficient because of the low spatial duty cycle, i.e., only a fraction of the entire volume is readout within a volume frame time. Even worse, repeated excitation of out-of-focus fluorescence, which is common in many scanning approaches, aggravates photobleaching and even photodamage.

Approaches for realizing parallelized illumination and detection in 3D imaging, i.e., all voxels are excited (recorded) simultaneously, become increasingly popular^2,7,8^. In addition to speeding up the imaging rate, parallelization in both the illumination and detection also maximizes the spatial duty cycle and consequently the photon budget. This is a critical requirement that ensures high-degree of viability of a biological sample. However, existing techniques of parallelization are either restricted to two dimensions (e.g., LSFM^3,7,8^) or sparse sampling in three dimensions (c.f., multi-focal^9–11^ or multi-light-sheet imaging^12–18^). In particular, the current multi-light-sheet fluorescence imaging systems, including the recent advances in lattice light-sheet microscopy, commonly resort to coherent beam interference, beam split, or wavefront shaping (in the spatial or Fourier domain)^12–15,19^. Yet, in order to minimize the illumination artifacts originating from the interplay between a coherent beam and highly scattering tissue (e.g., speckle noise), these strategies are limited to sparsely sampling the imaged volume with a handful of light sheets. Consequently, they necessitate serial beam scanning or dithering to effectively create a time-averaged superposition of incoherent light sheets^3^. Another emerging technique is light-field microscopy, in which the axial information can be distinguished via the structural dimensions on a 2D camera through an array of microlenses^20–22^. The final 3D image is computationally reconstructed with algorithms that solve the inverse problem, leading to the limitations of a reduced lateral resolution and a high computational complexity.

Here, we demonstrate a new strategy that exploits fully parallelized multiple-plane fluorescence imaging, called, coded light-sheet array microscopy (CLAM). Instead of relying on the common ideas of generating multiple coherent light sheets, e.g., dedicated wavefront control and precise mechanical beam scanning or dithering, CLAM achieves 3D parallelized illumination by harnessing the concept of “infinity mirror”. It makes use of the multiple reflections between an angle-misaligned mirror pair to generate a light-sheet array that is reconfigurable in both the array density and coherency. This approach enables an incoherent superposition of a dense and uniform light-sheet array (30–40 light sheets) without beam dithering, favouring deep and scattering tissue imaging with minimal illumination artifacts and speckle noise. Regarding the parallelized 3D image detection, CLAM implements multiplexed image-plane encoding that ensures optical sectioning without a scanning mechanism and thus allows fast volumetric frame rate. The 100% spatial duty cycle in the detection also implies a longer voxel dwell time. In other words, CLAM requires less intense illumination and thus further reduces the photodamage and photobleaching without sacrificing the signal-to-noise ratio (SNR). The concept of CLAM can easily be adapted to the existing LSFM systems with minimal hardware or software modifications (a complex iterative image reconstruction algorithm is not needed). To demonstrate the applicability of CLAM in practical biological imaging scenarios, we apply this fully parallelized 3D imaging technique to scattering media, optically cleared tissue structures, and microparticles in microfluidic flow at a volumetric frame rate above 10 vol/s.

## Results

### Working principle of CLAM

**Fig. 1:**
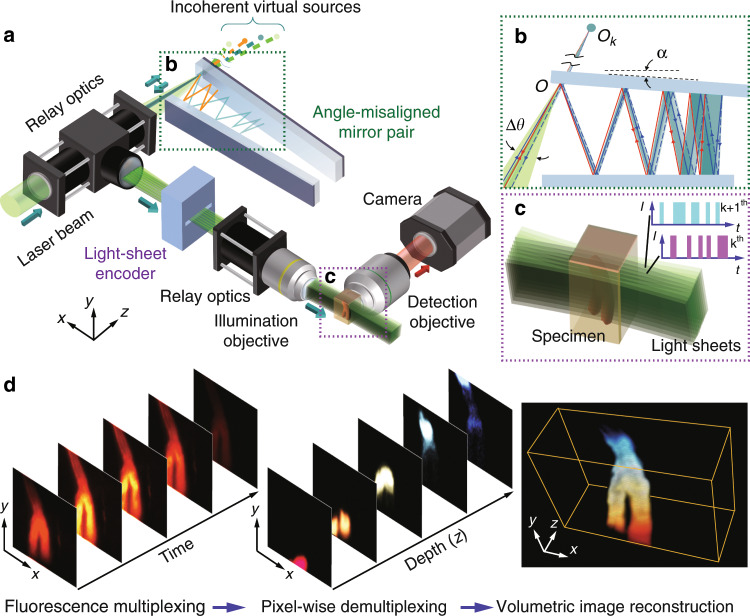
The concept of CLAM. **a** General schematic of the CLAM setup. The setup mainly consists of four key parts: (1) an angle-misaligned mirror pair (or “infinity mirror”) for the generation of the beamlet array; (2) a light-sheet encoder for the light modulation of the beamlet array with the temporal codes; (3) relay optics for shaping the beamlet array to the light-sheet array; and (4) imaging optics in which the detection arm and illumination arm are orthogonal to each other. **b** Virtual source generation (*O*_*k*_) by the “infinity mirror”. Using ray tracing, the kth beamlet decomposed from the incident light cone follows a total of 2*k* reflections between the angle-misaligned mirror pair and is retroreflected back to the entrance *O* (the solid line is the forward path, and the dotted line is the backward path). Furthermore, there is a light path, called the *cardinal mode/ray*, along which the forward and backward paths are overlapped (red rays). There are other sets of light paths within the *k*th beamlet satisfying the condition of 2*k* reflections. However, these beamlets follow slightly different trajectories after the entrance *O* (blue rays) such that their forward and backward paths are not overlapped. These light rays return to the entrance *O* but with a minute lateral shift from the cardinal ray. In effect, the *k*th *retroreflected* beamlet can be considered as a “light fan” diverging from a *virtual* source with a very low numerical aperture (≪0.1) located at *O*_*k*_. **c** Parallelized light-sheet array illumination. As each light sheet is temporally modulated with a unique code generated by the light-sheet encoder, the fluorescence signals from different depths along the z-direction, tagged with the same temporal codes, are multiplexed and detected by the camera. **d** Workflow of the image reconstruction of CLAM (for a volumetric image of a branching blood vessel).

Similar to typical LSFM, CLAM is configured here with the illumination and fluorescence detection paths orthogonal to each other (Fig. 1a). The unique feature that enables parallelized 3D imaging in CLAM is motivated by the concept of free-space angular-chirp-enhanced delay (FACED)^23–25^, which creates an ultrafast laser line-scanning action from multiple reflections of a pulsed laser beam between a pair of angle misaligned plane mirrors. In CLAM, we exploit this unique property to create an array of continuous-wave (CW) light sheets with reconfigurable density and coherency through flexible tuning of the mirror-pair geometry (e.g., the mirror length *L*, separation *S*, and misaligned angle *α*)^24^. In brief, a line-focused beam laser with a cone angle (Δ*θ*) illuminates a pair of nearly parallel high-reflectivity mirrors at the entrance *O* and is then split into a collection of *N* beamlets (*N* = Δ*θ/α*), with each beamlet following a unique zig-zag path between two mirrors. Because of the minute angle misalignment between the mirror pair, the mirror-reflection angles of each beamlet are progressively reduced, making the zig-zag path *spatially chirped*. Based on ray tracing, these *N* discrete light paths (called *cardinal modes*^24^) are retroreflected to the entrance *O* along the identical paths (e.g., the red path in Fig. 1b). On the other hand, the light paths deviating from a cardinal ray (e.g., the blue path in Fig. 1b) can still be routed back, but they have a minute lateral shift from the cardinal ray at the entrance *O*. In effect, for the *k*th returning cardinal ray, there is an accompanying “light fan” as if it has emerged (and diverged) from a *virtual* source with a very low numerical aperture (NA) (≪0.1) located at *O*_*k*_ (Fig. 1b). As a result, all the incoming rays within the incident light cone illuminating the mirror pair are retroreflected, transforming into an array of *N* discrete beamlets in analogy to rays emerging from an array of *N* virtual sources (Fig. 1a)^24^. The only optical loss is attributed to the mirror reflectivity, which is as high as >99.8% (see Methods). The virtual sources are projected through a relay-lens module to form an array of *N* light sheets (Fig. 1a, c and Supplementary Fig. S1). This approach enables parallelized 3D illumination and thus bypasses the need for beam or objective scanning, which requires dedicated synchronization. Our previous work showed that the number of light sheets (virtual sources) *N* is given by *N* = Δ*θ*/*α*. There is a maximum acceptance input cone angle *θ*_max_ within which all of the beamlets can be retroreflected without “leaking” away from the far end of the mirror pair. The acceptance angle is determined by the effective numerical aperture of the FACED mirror pair, given by $NA \approx \sqrt {L\alpha /2S}$^24^. In other words, the maximum number of light sheets supported by the mirror pair can be flexibly adjusted by the mirror geometry (e.g., *S*, *α*, *L*)^24^. We note that the number of light sheets is determined according to the imaging specifications (e.g., resolution, FOV, and photon budget). Here, *N* is chosen to be greater than 100 in the experiment. To ensure uniformity of the intensity, we further select the 30–40 highest-order cardinal modes for the CLAM illumination. Furthermore, the degree of coherence among the light sheets can flexibly be adjusted. While each light sheet itself remains coherent, the incoherency between the light sheets in the array can be achieved by tuning the mirror separation (*S*) in such a way that the path length difference (*D*) between the virtual sources (i.e., *D* *=* 2*S*) is longer than the coherence length of the laser source *L*_c_. The path length separation (temporal delay) between adjacent virtual sources can be reconfigured across several orders of magnitude, i.e., millimeters (picoseconds) to meters (nanoseconds)^24^. Such controllable degree of coherence minimizes the image artifacts and speckle generation, especially in a scattering medium.

Parallelized volumetric detection is accomplished by multiplexed light-sheet encoding. In general, the fluorescence signal from the *k*th section *I*_*em,k*_ (*x, y, z* − *z*_0_*k*) is intensity-modulated with a unique temporal code *m*_*k*_ (*t*) (Fig. 1c), with *z*_0_ being the adjacent light-sheet separation. The orthogonal detection objective collects the multiplexed fluorescent emission, which is registered on a 2D image sensor. The 2D raw data taken by the camera can be represented as (see Supplementary Information):

1 $$I_{{\mathrm{cam}}}\left( {x,y,t} \right) = {\sum \limits_{k = 0}^{N - 1}} \left[ {1 + m_k\left( t \right)} \right] \cdot {\int\nolimits_{ - \infty }^{ + \infty}} I_{em,k}\left( {x,y,z - z_0k} \right){\rm{d}}z$$

The image, with minimal cross-talk among the planes, can faithfully be recovered when the orthogonality of the codes is satisfied. Inspired by orthogonal frequency division multiplexing (OFDM) in wireless communication networks^26^, we modulate the light sheets with *m*_*k*_ (*t*) = cos(ω_*k*_*t*), where ω_*k*_ is the depth-dependent modulation frequency of the *k*th light sheet. The frequency carriers satisfy the orthogonality property over a period *T*, i.e., <*m*_*k*_(*t*),*m*_*j*_(t) > = *δ*_*ij*_, where *δ*_*ij*_ is the delta function, and <·> refers to an inner product. Therefore, 2D sections at different depths are tagged with distinguishable modulation frequencies and are multiplexed into a single 2D frame sequence registered on the image sensor (Fig. 1d). By applying a short-time Fourier transform on the 2D image sequence *I*_cam_ (*x, y, t*),

2 $$\tilde I_{{\rm{cam}}}\left( {x,y,\omega } \right) \propto \mathop {\sum }\limits_{k = 0}^{N - 1} \delta \left( {\omega - \omega _k} \right) \cdot I_{{\rm{em}},k}\left( {x,y} \right)$$

where $\tilde I_{{\rm{cam}}}\left( {x,y,\omega } \right)$ denotes the temporal Fourier transform of *I*_cam_ (*x, y, t*). In contrast to the existing frequency-multiplexed imaging approaches, which are adopted for 1D line illumination^27–30^, CLAM multiplexes 2D image stacks to enable parallelized 3D imaging. Among the available spatial light modulation techniques^27–31^, here, for simplicity, we adopt a spinning patterned reticle that provides a frequency-chirped intensity modulation across the light-sheet array (Supplementary Fig. S1)^30,31^.

The design rationale of the reticle pattern is generally guided by two key specifications that critically determine the CLAM performance (see Supplementary Information). First, the modulation frequency separation between adjacent light sheets (Δ*f*) defines the volumetric imaging rate (*f*_vol_), i.e., *f*_vol_ = Δ*f*. Furthermore, to ensure the best achievable axial resolution, Δ*f* should also be chosen such that the associated depth separation between the encoded frequency channel (i.e., Δ*d* = *β*Δ*f*, where *β* is the calibrated conversion factor between the depth and frequency) is kept equivalent or smaller than the thickness of each light sheet (*w*_LS_), i.e., Δ*d* < *w*_LS_. Second, governed by both the Nyquist sampling criterion and the camera frame rate, the total modulation frequency range (i.e., the bandwidth denoted by BW) used to encode all of the light sheets determines the number of light sheets (i.e., *N*). Following the Nyquist criterion, the upper limit of the modulation frequency (*f*_H_) should be less than half of the camera frame rate (*f*_cam_), i.e., *f*_H_ < *f*_cam_/2 (in fast mode, we set *f*_H_ ~ 1400 Hz < *f*_cam_/2). On the other hand, the lower limit of the modulation frequency (*f*_L_) should be greater than half of the upper frequency limit, i.e., *f*_L_ > *f*_H_/2, to eliminate the cross-talk from the high-order harmonic oscillations. For a given frequency bandwidth, i.e., BW = *f*_H_ − *f*_L_ (set by the design of the reticle and spinning speed), the number of frequency channels, or equivalently the number of light sheets (*N*), that can be allocated is *N* = BW/*δf* = BW/*f*_vol_. Hence, CLAM could faithfully generate *N* ≈ 20–70 light sheets to achieve a 3D imaging rate of *f*_vol_ = 1–20 vol/s. While such a volume rate is comparable to the volume rate of state-of-the-art scanning-based LSFM platforms and matches the speed required in many biological imaging applications, the multiplexing nature of CLAM further improves the sensitivity as all of the voxels in the volume are readout in parallel (i.e., 100% spatial duty cycle), increasing the effective voxel dwell time by a factor of the multiplexing number (*N*) without compromising the volume rate. Given this improvement, CLAM also reduces the illumination power and thus the photobleaching and phototoxicity. We note that multiplexing with CLAM introduces extra shot noise due to the cross-talk between the image stacks, whereas it also effectively distributes, and thus reduces, the readout noise in each encoded image stack. Therefore, CLAM improves the noise performance particularly when it operates in the readout-noise-limited regime. In the shot-noise-limited regime, the SNR of CLAM scales with the sparsity of the fluorescent sample with the caveat that multiplexing inherently distributes the shot noise across all of the 2D stacks (see Supplementary Materials).

### CLAM performance

**Fig. 2:**
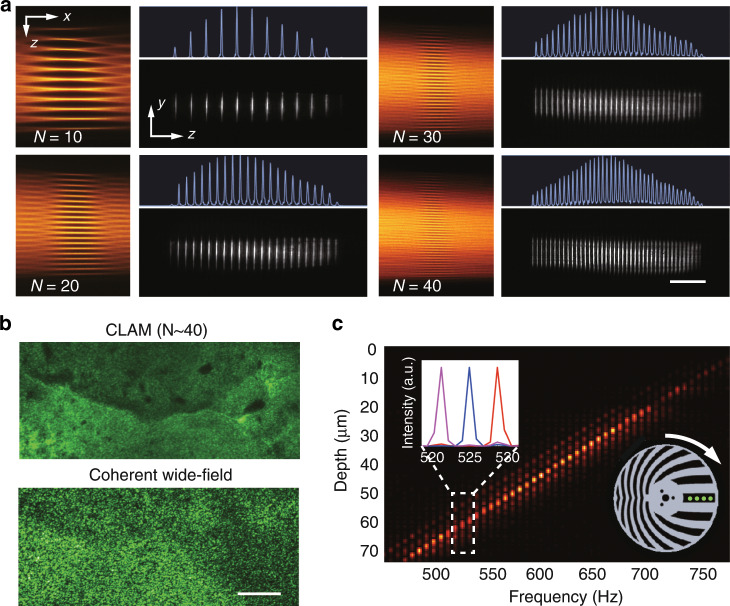
System performance of CLAM. **a** Measured transverse profiles (in the *x*-*z* and *y*-*z* planes) of the light-sheet arrays (*N* = 10, 20, 30, and 40). The corresponding intensity line-scan profile of each case is shown in blue. **b** Images of a light-scattering phantom (agarose gel embedded with titanium dioxide (*TiO*_2_) nanoparticles) obtained by (top) incoherent CLAM beams (*N* = 40) and (bottom) coherent wide-field illumination. **c** The frequency-to-depth map shows the linear relation between the encoding frequencies and the depth (*N* = 40). Minimal cross-talk is observed between individual light sheets (frequency channels) (upper left inset). (Lower inset) Schematic of the spinning reticle (i.e., light-sheet encoder) and the projected virtual sources (green dots). The scale bars represent 50 μm.

In comparison to other LSFM techniques, CLAM generates an array of light sheets, for which the incoherency and light-sheet density can be flexibly reconfigured by adjusting the geometry of the angle-misaligned mirror pair. In particular, the density or the number of light sheets can be selected from *N* = 10 to *N* = 40 by adjusting the angle-misalignment between the mirror pair *α* (Fig. 2a and Supplementary Fig. S3). Both simulation and experiment show that the light-sheet array exhibits high uniformity in the sheet thickness across the whole 3D FOV, regardless of the number of light sheets (Fig. 2a and Supplementary Fig. S4).

Furthermore, the inter-sheet incoherency in CLAM can be tailored by controlling the spatiotemporal separation and thus the path length difference (*D*) between the neighboring virtual sources to be greater than the coherence length of the laser source *L*_c_. Such incoherency is manifested by the largely smooth illumination distribution without observable interference when the light-sheet density is increased such that individual sheets are overlapped (Fig. S3). This scenario is consistent with the fact that the virtual source separation (*D* ~ 40 mm) is larger than the measured coherence length of the light source (*L*_c_ ~ λ^2^/ Δλ = 0.1 mm as measured from the laser spectrum with a bandwidth of Δλ ~ 5 nm and center wavelength of λ = 712 nm) (see Supplementary Materials). The incoherency was further validated in another configuration, which employed a laser source with a center wavelength of 532 nm and a bandwidth of 44.5 pm, resulting in a coherence length of *L*_c_ ~ 4.2 mm ≪ *D* ~ 100 mm (see Methods). This configuration exhibits a speckle-free light-sheet array illumination distribution through a light-scattering phantom embedded with nanoparticles (see Methods). This result is in clear contrast to the case of wide-field coherent illumination of the same region, expanded from a single coherent Gaussian laser beam, resulting in highly speckled patterns (Fig. 2b).

In the absence of an emission filter, the frequency-encoded light sheets directly reflect off the glass slide and are captured by the sCMOS camera in the orthogonal detection arm. This setup allows the evaluation of the frequency encoding characteristics. By applying a short-time Fourier transform to the temporal signal pixel by pixel, we generate a frequency-depth map that shows a clear linear relationship (*R*^2^ = 0.995, with a slope of *β* = 0.23 μm/Hz) between the encoded depth and the modulation frequency (*N* = 40) (Fig. 2c). This observation is in accordance with the reticle design, which generates a linear frequency chirp across the beamlet array (see Supplementary Information)^30,31^. Individual decoded light sheets, with an average thickness of ~1.5 μm (Fig. 2c), are tagged with distinctive center frequencies between 450 Hz and 750 Hz and are consistently associated with the finite bandwidth (~3 Hz) (Fig. 2c). The average SNR over all frequency channels is >5 dB with minimal cross-talk between neighboring channels, as corroborated in the frequency-depth map (Fig. 2c). We note that the residual side lobes in each frequency channel are mainly attributed to the mechanical jitter and wobble of the spinning reticle.

**Fig. 3:**
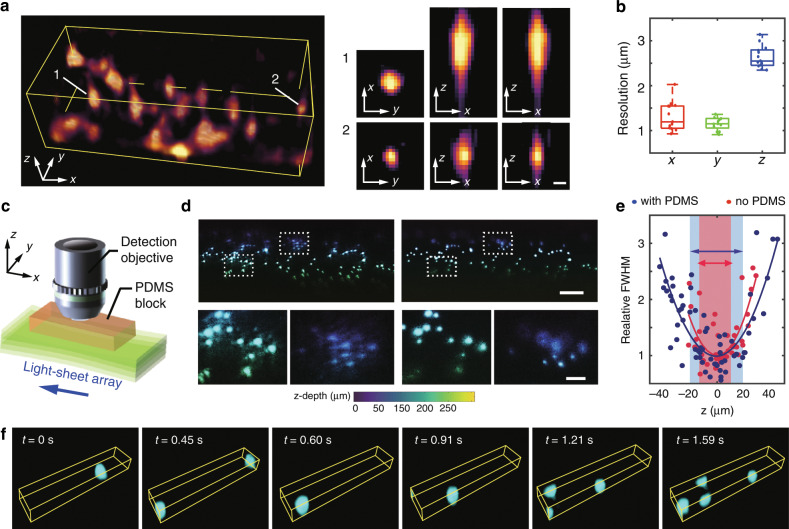
Evaluation of the point spread function (PSF) of CLAM. **a** (Left) CLAM images of the nanobeads (dispersed on a tilted cover slide) for the PSF measurement (volume of 90 × 40 × 25 μm^3^); (right) the projected 2D PSFs measured from two beads (beads 1 and 2). The scale bars are 2 μm. **b** Statistics (n = 13) of the measured resolutions along the three dimensions (represented in boxplots) within the volume of 90 × 40 × 25 μm^3^. **c** Implementation of the extended depth of field (DOF) based on the induced spherical aberration, which is implemented by placing a PDMS block between the sample (a tilted cover slide with dispersed nanobeads) and the detection objective. **d** Comparison of the DOFs between the CLAM images obtained with and without the PDMS block. The depth is color coded. The scale bars represent (top) 50 μm and (bottom) 20 μm. **e** Measured full-width-half-maximum (FWHM) (relative to the minimal value) of the PSF along the depth (*z*) in the cases (red) without and (blue) with the PDMS block. The solid curves are a quadratic fit. The DOF is extended by ~32% in the presence of spherical aberration. **f** Imaging of microparticles flowing in the micropipette with a volumetric frame rate of 13.2 vol/s (volume of 170 × 25 × 28 μm^3^).

The image reconstruction in CLAM simply adopts the short-time Fourier transform of the frequency-multiplexed image data pixel by pixel, followed by standard Richardson–Lucy deconvolution. The 3D point spread function (PSF) of CLAM was evaluated by imaging fluorescent spheres, with a diameter of 100 nm, coated on a cover slide mounted at 45° with respect to both the illumination and detection arms. The measured transverse resolution (full-width at half-maximum, FWHM) of ~1.2 μm, approaches the diffraction limit (NA = 0.25), whereas the axial resolution (~2.7 μm) (Fig. 3a, b) is determined by the thickness and separation of light sheets, as well as the optical transfer function of the detection objective, especially in the presence of spherical aberration, which is introduced to extend the depth of field (DOF) (as described below)^8^.

We further harness the spherical aberration to expand the DOF^32^ in order to maximize the advantage of parallelized array illumination. This step is made possible by introducing a block of high-refractive-index polydimethylsiloxane (PDMS, refractive index of *n* = 1.42 and thickness of 5 mm) mounted between the detection objective and the sample (Fig. 3c). In the presence of a high-refractive-index medium, fluorescent emission light rays, especially peripheral rays, will be focused at different points along the depth axis, thereby creating a sizable but uniform spherical aberration. This aberration effectively extends the DOF without significantly degrading the transverse resolution (Fig. 3d). We experimentally measured the FWHM of the PSFs across the FOV for the two cases, i.e., with and without an extended DOF (Fig. 3e). In comparison, the PSF without an extended DOF broadens more rapidly along the axial direction. Defining the DOF as the range within which the FWHM is maintained within the $\sqrt 2$-fold minimum achievable FWHM, we observe that the current configuration could yield a ~32% extension of the DOF, i.e., the axial FOV is extended from 31 μm to 41 μm (Fig. 3e). In contrast to PSF engineering techniques involving a non-diffracting illumination beam, e.g., using an axicon or tunable acoustic gradient-index lens^5,33^, this method is scalable in the DOF, allowing one to tailor different experimental specifications by tuning the refractive index without a dedicated optical system alignment.

We further evaluated the imaging speed of CLAM by imaging flowing fluorescent beads supplied by a microfluidic pump (Harvard, Phd 2000) into a fluidic channel (square glass pipette with an inner side length of 1 mm). In this proof-of-principle demonstration, we configured the CLAM system with a total of *N* = 24 light sheets within the frequency range of 1.1–1.4 kHz. This system is able to visualize the flowing microspheres (flow rate of ~20 µm/s) at a volumetric rate *f*_vol_ of up to 13 vol/s (Fig. 3f). We note that the *practical* volume rate in the current setup can further be enhanced depending on the number of light sheets (*N*) required for the experiments. For instance, the volume rate can be increased to ~25 vol/s with our current camera when the imaging FOV along the axial direction is reduced by half (i.e., *N* = 12). Furthermore, as the volume rate achievable in CLAM is only limited by the camera speed (currently limited at ~1000–3000 fps in our system), we anticipate that the volume rate can readily be scaled beyond 100 vol/s with a state-of-the-art high-speed intensified camera (>10,000 fps)^34^.

**Fig. 4:**
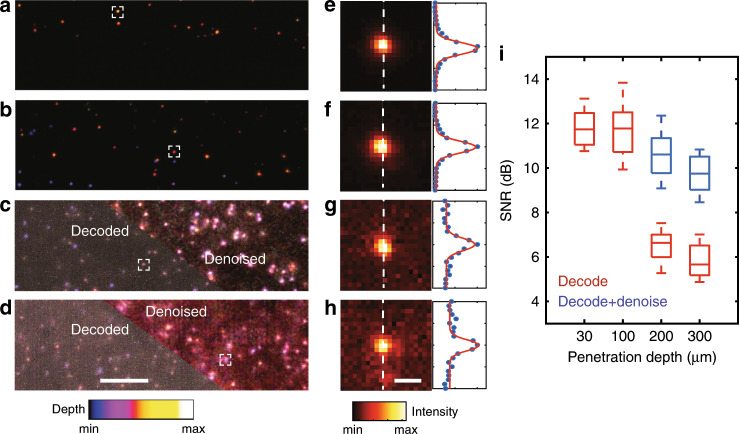
Volumetric CLAM imaging of light-scattering fluorescence microbeads embedded in agarose gel. The imaging depths are (**a**, **e**) 30 μm, (**b**, **f**) 100 μm, (**c**, **g**) 200 μm, and (**d**, **h**) 300 μm; the top right half of the images in (**c**, **d**) are denoised for a comparison. The depth is color-coded in (**a**–**d**). **e**–**h** show the maximum intensity projections (left) and linear intensity profiles (right) of the beads marked in (**a**–**d**). **i** SNR as a function of the penetration depth. Image denoising is applied for a comparison at the deeper depth. The scale bars represent 40 μm (**a**–**d**) and 5 μm (**e**–**h**).

We next investigated the performance of CLAM in a scattering medium at different penetration depths. For a verification, we imaged fluorescence microbeads (diameter of 1 µm) embedded in a tissue-mimicking phantom. The fluorescence profiles of the microbeads are consistent for depths up to 300 μm without severe distortion (Fig. 4a–d). An inspection of the transverse and linear profile of an individual nanosphere image allows a dissection of the image quality (Fig. 4e–h). The SNR of the raw image can be maintained at 5 dB at a depth of 300 μm and is readily enhanced to 9 dB through standard denoising (Fig. 4i). The titanium dioxide (TiO_2_) nanoparticle in the phantom has an average diameter of 160 nm as characterized by transmission electron microscopy. A Mie scattering calculation suggests a reduced scattering coefficient of ${\upmu}_{\rm{s}}^\prime \sim 22\,{\rm{cm}}^{ - 1}$ (i.e., a transport mean free path of ~450 μm) at 532 nm (see Supplementary Materials), which is comparable to the reduced scattering coefficients of biological tissue^35^. This result is consistent with the fact that the captured image at a depth of 300 μm maintains a reasonable SNR of ~6 dB (before denoising) (Fig. 4). Such performance suggests that 3D parallelized illumination using a dense, incoherent light-sheet array and thus multiplexed light-sheet coding do not compromise the penetration depth in a highly scattering medium, which is comparable to the state-of-the-art scanning-based confocal and LSFM modalities (~100 μm in depth).

### Volumetric imaging of tissue cleared structures

We next sought to apply CLAM to 3D visualization in combination with tissue clearing—a powerful strategy that enables imaging of thick tissues and even whole organisms by homogenizing the refractive index throughout the specimen without altering its anatomical structure through chemical treatments. This approach is particularly valuable for minimizing light scattering in tissues and thus enhancing the image quality of thick tissues^36^. Here, we adopt a recent method, called OPTIClear, because of its detergent- and denaturant-free nature with minimal structural and molecular alteration^37,38^. OPTIClear is also compatible with a wide range of fluorescent dyes and both fresh and archival samples, including both human and small animal (e.g., rodent) tissues. We employed CLAM to image OPTIClear-treated mouse ileum and kidneys that were labeled with a lipophilic carbocyanine dye (DiI)^39^. The specimen was immersed in a medium (*n* = 1.47) to achieve a spherical-aberration-assisted extended DOF.

**Fig. 5:**
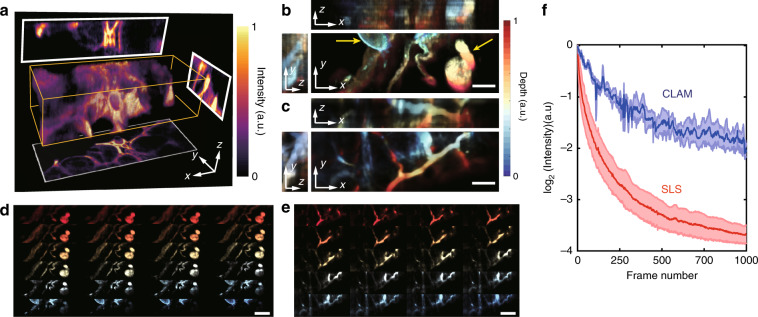
Volumetric imaging of optically cleared mouse tissue. **a** 3D rendered image of the tubular epithelial structures in the mouse kidney (*N* = 34) at a volume rate of 6.6 vol/s (with three orthogonal standard deviation intensity projections (SDIPs)). The volume is 230 × 73 × 65 μm^3^. The SDIP of (**b**) the glomeruli (indicated by arrows) and (**c**) the intestine blood vasculature in the mouse along the three orthogonal axes. **d**, **e** Montages of 2D sections of the images in (**b**, **c**), respectively. **f** Comparison of the photobleaching for two illumination conditions: a single light sheet (SLS, *N* = 1, red) and a light-sheet array (CLAM, *N* = 40, blue). The shaded areas represent ±1 standard deviation for each condition (20 and 30 measurements for the cases of SLS and CLAM, respectively). The scale bars represent (**b**, **c**) 20 µm and (**d**, **e**) 40 µm.

We demonstrate that tubular epithelial structures (Fig. 5a), the glomeruli (Fig. 5b), and the intestine blood vasculature (Fig. 5c) can all be imaged at a volume rate of ~6.6 vol/s. Here, CLAM collects all of the optically sectioned images simultaneously using 34 multiplexed and densely packed light sheets (Fig. 5d, e). More importantly, the applicability of CLAM in biological imaging is further substantiated by its much lower risk of photodamage and/or photobleaching due to 3D parallelization. We demonstrate this benefit by comparing the photobleaching effects of two illumination scenarios corresponding to LSFM and CLAM (see Methods). Clearly, CLAM outperforms LSFM with significantly slower photobleaching (Fig. 5f). The lower photobleaching rate and thus potentially lower risk of photodamage/phototoxicity in CLAM is attributed to its highly parallelized operation (100% spatial duty cycle), which requires a lower illumination intensity and allows a longer exposure time^40^ without sacrificing the SNR and the imaging speed. Therefore, we anticipate that CLAM could be particularly valuable in long-term biological monitoring applications, especially in developmental biology.

## Discussion

We have demonstrated a new volumetric imaging platform, CLAM, that exploits a complete parallelized 3D multiplexed LSFM strategy. In contrast to the existing LSFM modalities, the defining feature of CLAM is its generation of a dense light-sheet array (>30), which is reconfigurable in both temporal coherency and spatial density. Furthermore, the 3D imaging throughput and efficiency can be maximized (spatial duty cycle of 100%) by multiplexed light-sheet coding (i.e., OFDM). The combination of these two features enables the simultaneous capture of all-optically sectioned image planes in a continuous volume at >10 vol/s, obviating the need for bulky objective scanning or beam scanning/dithering. CLAM does not have a fundamental limitation in scaling to a higher volume rate as sCMOS technology continually advances (e.g., >10,000 fps in a state-of-the-art sCMOS camera)^34^. More importantly, CLAM simultaneously reads out all of the voxels in three dimensions and thus allows a longer integration (exposure) time and potentially better SNR, especially for sparse samples. CLAM thus reduces the illumination power and outperforms other LSFM modalities in reducing the photobleaching/phototoxicity. CLAM only replaces the beam scanning module by the mirror pair (for parallelized illumination) and the spinning reticle (for parallelized encoding). Hence, CLAM can readily be compatible with any existing LSFM modalities with minimal hardware modification or dedicated synchronization. In this regard, CLAM could easily be further advanced to tailor specific applications. For instance, parallelized discrete light-sheet array illumination also provides another degree of freedom to arbitrarily select subsets of light sheets. This could be of particular interest in sparse sampling of neuronal activity recording in brain imaging applications^1,2^. Simultaneous multi-view CLAM can also be implemented by illumination with the light-sheet array from multiple directions—an effective strategy that has been proven to improve the image quality in the presence of light scattering and resolution isotropy^41^. CLAM should also be compatible with the available wavefront coding/shaping techniques used for increasing the FOV in both the axial and lateral dimensions^4,42^. Notably, CLAM, when combined with additional spatial light modulation and/or a beam scanning module, can be adopted to create a more sophisticated structured illumination, such as a lattice light sheet^12,43,44^. CLAM can also be combined with an adaptive optics module to overcome image distortion and aberration in deep tissue in vivo^11,45^, which could impact applications in neurobiology.

In conclusion, CLAM fully parallelizes 3D illumination and detection within the imaging FOV that is otherwise missing in the current LSFM modalities. This feature, requiring straightforward software and hardware configurations, makes CLAM an efficient volumetric imaging approach in terms of both photodamage/phototoxicity and speed. CLAM could thus be pertinent to the unmet needs in neuroscience and developmental biology research for effective tools that enable dynamical imaging of live cells, tissue, and organisms in long-term and large-scale examination of archival (cleared) biological samples.

## Materials and methods

### Experimental setup

The collimated beam from a diode-pumped solid-state laser (CW, wavelength of 532 nm, power of 400 mW, Shanghai dream laser) was line-focused by a cylindrical lens (*f*_CL_ = 200 mm) into an angle-misaligned mirror pair (reflectivity of *R* > 99.8%, separation of *S* = 50 mm, length of *L* = 200 mm, IOS optics) at the entrance *O*. The beam breaks into a discrete set of (spatially chirped) zig-zag paths governed by their incident angles. The number of beamlets *N* was mainly controlled by the misalignment angle, the light cone angle and a variable slit (*N* was chosen to range from 30 to 70). This beam was collected by a lens (*f* = 200 mm) and relayed through a telescope T2 (2x magnification) onto the spinning reticle (i.e., the light-sheet-array encoder based on OFDM), followed by another telescope T3 (1/4 magnification) to match the FOV. All of the virtual sources were imaged in planes in proximity to the common focal plane (CFP) of L and T2^24^. This configuration essentially ensures that all of the virtual sources are imaged within the DOF of the illumination objectives (Fig. S1). The beam passes an illumination tube lens TL1 (*f* = 200 mm) and a cylindrical lens CL2 (*f* = 50 mm) and then is focused by an illumination objective O1 (20×, NA = 0.45, W.D. of 8.2–6.9 mm, Nikon) to generate the light-sheet array. The orthogonal detection objective O2 (10×, NA = 0.25, W.D. of 10.6 mm, Olympus) collects the multiplexed fluorescent emission, which is registered on a 2D image sensor (pixel size of 6.5 μm, Andor Neo 5.5) operating in rolling shutter mode with a frame rate up to 3183 fps. A long-pass filter (cut-off at 543 nm, Chroma) in the detection arm cleans the excitation light (see Supplementary Fig. S1). The frequency reticle has a transmission function of $T\left( {r,\varphi } \right) = \frac{1}{2} + \frac{1}{2}{\mathop{\rm{sgn}}} \left[ {{\mathrm{cos}}\left( {\omega \varphi } \right)} \right]$, where *ω* = 2*πr* is the radius-dependent modulation frequency and sgn() is the sign function (Fig. 2c and Supplementary Fig. S1). The reticle was fabricated on a transparency film (diameter of 120 mm) and was spun by a step motor (rotation speed of 120–2000 rpm) with phase-locked angular-speed control (MC2000B, Thorlabs). To evaluate the photobleaching performance, we configured the setup to generate three illumination scenarios corresponding to CLAM, a single-beam light sheet, and confocal microscopy. For the single-beam light sheet and confocal cases, the coherent illumination bypasses the mirror pair with additional (cylindrical) lenses to manipulate the incident profile.

### Sample preparation

Fluorescent polymer nanospheres (F8800, 100 nm, Life Technology) were dispersed on a standard coverslip, mounted at 45° with respect to both the illumination and detection paths (Fig. 3). This configuration allowed us to calibrate the encoded depth, evaluate the PSF across the 3D FOV and characterize the performance of the spherical-aberration-induced extended DOF. The DOF was introduced by placing a block of PDMS (with a refractive index of *n* = 1.42 and thickness of 5 mm) between the detection objective and sample. To evaluate the imaging performance in a scattering medium, we imbedded fluorescent polystyrene beads (F8819, 1 μm, Life Technologies Ltd.) into 2% agarose gel mixed with *TiO*_2_ nanoparticles (average diameter of 160 nm; see the TEM images in Supplementary Fig. S5). The concentration of TiO_2_ nanoparticles was chosen as 1.2 mg/mL to provide a scattering strength similar to that of biological tissues^35^, which have a typical reduced scattering coefficient ranging between ${\upmu}_{\mathrm{s}}^\prime \sim$ 10−100 cm^−1^. All of the animal work was approved and handled in accordance with the guidelines provided by the Committee on the Use of Live Animals in Teach and Research (CULATR) in the Laboratory Animal Unit, HKU with approval reference no. 3699-15. One adult C57BL/6 mouse was euthanized using intraperitoneal sodium pentobarbital (150 mg/kg), transcardially flushed with normal saline, and perfusion-fixed with freshly prepared 4% phosphate-buffered paraformaldehyde. The lipophilic tracer DiI (Invitrogen D282) was then administered transcardially^39^. Approximately 3-cm-long segments of ileum and whole kidneys were harvested after the mouse tissue was dissected, cut into 2-mm-thick slices, and stored in 1× phosphate-buffered saline at 4 °C. The tissue blocks were immersed in OPTIClear^37,38^ and incubated at 37 °C overnight.

### Image processing

A short-time Fourier transform of the 2D raw multiplexed data over a period of 1/*f*_vol_ yields a set of *N* frequency comb lines, with each line carrying an amplitude of *I*_em*,k*_ (*x, y*), i.e., the total emission signal projected from the *k*th light sheet (see Supplementary Information). This operation directly produces the entire 3D information in parallel with the frequency axis mapped to the depth of the imaged volume (see the frequency-depth calibration in Fig. 2c). We further performed digital smoothing in the frequency (axial) domain using a boxcar filter during the image reconstruction, removing the potential artifact produced by the discrete frequency channels (light sheets) at the expense of axial resolution. This step, together with the presence of spherical aberration (which is intentionally introduced to extend the DOF), determines the final axial resolution of the system. The reported resolution refers to the values extracted from raw images for the nanospheres. We applied a nonlinear iterative Richardson–Lucy deconvolution to the Fourier-transformed image data (10–40 iterations implemented in MATLAB, MathWorks Inc.) to further eliminate the image blurring owing to a finite exposure time. The deconvolved image was then passed to Fiji (ImageJ) for 3D image rendering^46,47^.

### Photobleaching test

We compared the photobleaching of CLAM and LSFM in the same system (shown in Supplementary Fig. S1), except that the beam was normally incident onto mirror M1 in the LSFM case to generate a single light sheet. Hence, the dimensions of the individual light sheets generated in both operation modes are identical (i.e., sheet thickness of ~3 μm and sheet height (in the *y*-direction) of ~50 μm). Both modes share the same confocal parameter *b* along the *x*-direction (i.e., *b* ~ 50 μm), and imaged the same sample of breast cancer cells, MB-231 (labeled with DNA dye for live cells, Vybrant™ DyeCycle™ Orange), on a glass slide (similar to the configuration shown in Fig. 3, but without the PDMS block that introduces the spherical aberration). The power levels for each mode were adjusted to achieve a similar signal (photon count read by the camera). The exposure times for each mode were also tuned such that they both had the same volume rates, i.e., in CLAM, the exposure time was 77 ms for *N* = 27. In LSFM, the exposure time for each light sheet was 2.9 ms, considering the same number of light sheets (*N* = 27) being scanned for the same volume. In both cases, we evaluated the average intensity in the region of interest (10 measurements for each case) within each labeled cell nucleus.

## Supplementary information

Supplementary Information for Parallelized volumetric fluorescence microscopy with a reconfigurable coded incoherent light-sheet array

## Data Availability

All the data supporting our findings are presented in the main text and the supplemental document. The MATLAB codes are available from the corresponding author upon reasonable request.
