# Supplementary material for: Parallelized volumetric fluorescence microscopy with a reconfigurable coded incoherent light-sheet array
Source: Light Sci Appl. 2020 Jan 20;9:8. doi: 10.1038/s41377-020-0245-8 (PMC6971027; doi:10.1038/s41377-020-0245-8)
Supplement: Supplementary file 1 — Supplementary Information for Parallelized volumetric fluorescence microscopy with a reconfigurable coded incoherent light-sheet array [file 41377_2020_245_MOESM1_ESM.pdf]

Supplementary material for

Parallelized volumetric fluorescence microscopy with a reconfigurable coded incoherent light-sheet array

Yu-Xuan Ren[1,*], Jianglai Wu[1,2,*], Queenie T. K. Lai[1,*], Hei Ming Lai[3,4], Dickson M. D. Siu[1], Wutian Wu[3,5,6], Kenneth K. Y. Wong[1], Kevin K. Tsia[1]

[1]*Department of Electrical and Electronic Engineering, The University of Hong Kong, Hong Kong SAR, China;*

[2]*Department of Physics, University of California, Berkeley, California 94720, USA*

[3]*School of Biomedical Sciences, Li Ka Shing Faculty of Medicine, The University of Hong Kong, Hong Kong SAR, China*

[4]*Department of Psychiatry, Faculty of Medicine, The Chinese University of Hong Kong, Hong Kong SAR, China*

[5]*GHM Institute of CNS Regeneration, Jinan University, 601 Huangpu Avenue West, Guangzhou 510632, China*

[6]*Re-Stem Biotechnology, Suzhou, China*

Correspondence to KT (tsia@hku.hk)

*These authors made equal contribution.

1. General theoretical framework

In general, CLAM can adopt any set coding functions $\{m_k(t)\}$, where k = {0, 2, ..., N-1}, given that they are orthogonal to each other over a period T, i.e., < $m_k(t)$, $m_j(t)$ > = 0, where k≠j and d< > refers to the inner product over the interval T: $\int_0^T m_k(t)m_j(t)dt$. In this work, we adopted OFDM, i.e., $m_k(t) = \cos(\omega_k t)$. Hence, individual light sheets are modulated by unique frequency carriers which are orthogonal to each other, because of the simple modulation implementation (simply by the spinning patterned reticle), its resilience to cross-talk among frequency carriers and its high spectral efficiency (i.e., it allows closely-spaced carriers within a given available modulation frequency bandwidth (BW))[1]. The modulation is done by temporal intensity-modulation of the beamlets by a rotating reticle, positioned at the conjugate plane of the virtual sources. The reticle was made of a transparency film printed with a circular mask pattern (a diameter of 120 mm) (**Fig. S1**), defined by a transmission function, $T(r,\varphi) = \frac{1}{2} + \frac{1}{2}\text{sgn}[\cos(\omega\varphi)]$, where $\omega = 2\pi r$ is the radius-dependent modulation frequency, and sgn() is the sign function. This gives rise to a linear chirp in modulation frequency along the radius. As the spot size of each beamlet is much smaller than the radius of

the reticle, we could neglect the minute range of modulation frequencies within each beamlet. Thus, the kth beamlet, and thus the kth light sheet, is regarded to be modulated simply by a single tune frequency.

The enabling feature in CLAM is the angle-misaligned mirror pair which generates spatially separated and incoherent virtual sources. Hence, this allows the incoherent superposition of the light-sheets for parallelized 3D illumination. Therefore, the intensity profile of the illumination can be given by:

$$I_{illum}(x,y,z,t) = \sum_{k=0}^{N-1} I_{sheet}(x,y,z-z_0k)\cdot[1+\cos(\omega_k t)], \tag{1}$$

where $I_{sheet}(x,y,z-z_0k)$ is the intensity spatial profile of individual light sheet, which can be modeled as any common light-sheet forms, such as Gaussian and Bessel beams, z_0 is the adjacent light-sheet separation. In this work, we form, from the virtual sources, the Gaussian light sheet, i.e., $I_{sheet}(x,y,z-z_0k) = I_0\left(\frac{\omega_0}{\omega(x)}\right)^2 \exp\left(-\frac{2z^2}{\omega(x)^2}\right)$, ω_0 and $\omega(x)$ are the beam waist of the light-sheet and the varying "thickness" of the light sheet along the illumination axis (x-direction), respectively. I_0 is the peak intensity. The fluorescence emission from different depth would then be modulated by the frequency ω_k corresponding to the kth light sheet. Hence, the multiplexed intensity profile captured by the 2D image sensor is given by:

$$\begin{aligned} I_{cam}(x,y,t) &= \int_{-\infty}^{+\infty}\sum_{k=0}^{N-1} I_{sheet}(x,y,z-z_ok)\cdot\left[1+\cos(w_k t)\right]\cdot O(x,y,z,t) * h_{\text{det}}(x,y,z)\,dz \\ &= \sum_{k=0}^{N-1}\left[1+\cos(w_k t)\right]\cdot\int_{-\infty}^{+\infty} I_{sheet}(x,y,z-z_ok)\cdot O(x,y,z,t) * h_{\text{det}}(x,y,z)\,dz \\ &= \sum_{k=0}^{N-1}\left[1+\cos(w_k t)\right]\cdot\int_{-\infty}^{+\infty} I_{em,k}(x,y,z-z_ok)\,dz \end{aligned} \tag{2}$$

where $O(x,y,z,t)$ is the imaged volume and $h_{det}(x,y,z)$ is the point spread function (psf) of the detection objective, including the spherical aberration effect induced for extended DOF. Here, we further define $I_{em,k}(x,y,z-z_ok)$ as the convolved emission signal generated from the kth light sheet. Taking the short-time Fourier transform over the period $T = 1/f_{vol}$ (i.e., the volume frame time), we obtain,

$$\begin{aligned} F\{I_{cam}(x,y,t)\} &= \tilde{I}_{cam}(x,y,\omega) \\ &= F\left\{\sum_{k=0}^{N-1}\left[1+\cos(\omega_k t)\right]\cdot\int_{-\infty}^{+\infty} I_{em,k}(x,y,z-z_ok)\,dz\right\} \\ &= F\left\{\sum_{k=0}^{N-1} I_{em,k}(x,y) + \sum_{k=0}^{N-1}\left[\cos(\omega_k t)\cdot I_{em,k}(x,y)\right]\right\} \\ &= A\cdot\delta(0) + \sum_{k=0}^{N-1}\delta(\omega-\omega_k)\cdot I_{em,k}(x,y) \end{aligned} \tag{3}$$

$F\{\cdot\}$ is the Fourier transform operator. A is the amplitude of the DC component. Eq. (3) indicates that the Fourier transform of the 2D raw multiplexed data yields a set of N frequency comb lines, each of which carries

the amplitude of $I_{em,k}(x,y) = \int_{-\infty}^{+\infty} I_{em,k}(x,y,z-z_0 k)dz$, i.e., the total emission signal projected from the kth light sheet onto the 2D image sensor. In other words, this operation directly results in the entire 3D information in parallel with the frequency axis mapped to the depth of the imaged volume.

2. Noise analysis

As CLAM captures multiple 2D image stacks in parallel, this inherently increases the dwell time (or integration time) of each voxel for a given volume frame rate. Hence, compared to the laser-scanning based LSFM, CLAM requires less intense illumination (and thus results in less photodamage/photobleaching) for achieving the same level of SNR. In other words, under the same illumination intensity for both CLAM and laser-scanning LSFM, CLAM yields an improved SNR. Here we present a noise analysis of CLAM, which primarily focuses on the effect of photon shot noise, which is the dominant noise source in CLAM for many 3D imaging applications. We first evaluate the average power S_k captured from each pixel on camera at the kth frequency channel over the volume frame time $T_{vol} = 1/f_{vol}$,

$$S_k = \int_0^{T_{vol}} m_k(t)dn(t). \tag{4}$$

In practice, the frequency chopper switches the beamlets on and off, the modulation code for OFDM, should be represented as $m_k(t) = 1 + \cos(\omega_k t)$, the fundamental oscillation satisfies the orthogonality condition. Here we define $dn(t)$ to be the total number of photons captured from the volume, consisting of N 2D stacks within an infinitesimal time dt (**Fig. 1**). Following Poisson statistics, $dn(t)$ is an independent random variable with the mean μ_n and the variance σ_n^2 given by

$$m_n = s_n^2 = \sum_{k=0}^{N-1}\left[1+\cos\left(w_k t\right)\right]\frac{\bar{n}_k}{T_{vol}}\cdot dt \tag{5}$$

where $\bar{n}_k$ is the average photon number from the kth 2D stack (frequency channel) within the volume frame period T_{vol}. Based on Eq. (5), S_k is regarded as a random variable which describes the signal captured from the kth stack, with the mean μ_{S_k} given by:

$$
\begin{aligned}
\mu_{S_k} &= \left\langle \int_0^{T_{vol}} m_k(t)\,dn(t) \right\rangle \\
&= \int_0^{T_{vol}} \left[1+\cos(\omega_k t)\right] \sum_{j=0}^{N-1} \left[1+\cos(\omega_j t)\right] \frac{\bar{n}_j}{T_{vol}} \cdot dt \\
&= \frac{1}{T_{vol}} \sum_{j=0}^{N-1} \bar{n}_j \int_0^{T_{vol}} \left[1+\cos(\omega_k t)+\cos(\omega_j t)+\cos(\omega_k t)\cos(\omega_j t)\right] \cdot dt \\
&= \sum_{j=0}^{N-1} \left(\bar{n}_j + \frac{\bar{n}_j \delta_{kj}}{2} \right) \\
&= \bar{n}_{vol} + \frac{\bar{n}_k}{2}
\end{aligned} \qquad (6)
$$

$\bar{n}_{vol}$ is the average photon number from all N stacks over one volume frame period. This is essentially equivalent to the background DC term ($\omega = 0$) in Eq. (3), which is filtered out during image reconstruction. Therefore, the resultant average signal should be $S_k \approx \overline{n_k}/2$. Note that the orthogonal property of the frequency carriers is applied in Eq. (6). This is represented by the Kronecker delta δ_{kj}, i.e., $\delta_{kj}= 0$ when $k \neq j$ and $\delta_{kj}= 1$ when $k = j$. The variance of S_k (${\sigma_{S_k}}^2$) is on the other hand given by,

$$
\begin{aligned}
{\sigma_{S_k}}^2 &= \int_0^{T_{vol}} Var\left\{ m_k(t)\,dn(t) \right\} \\
&= \int_0^{T_{vol}} \left[1+\cos(\omega_k t)\right]^2 \sum_{j=0}^{N-1} \left[1+\cos(\omega_j t)\right] \frac{\bar{n}_j}{T_{vol}} \cdot dt \\
&= \frac{1}{T_{vol}} \sum_{j=0}^{N-1} \bar{n}_j \int_0^{T_{vol}} \left[1+\cos^2(\omega_k t)+2\cos(\omega_k t)\cos(\omega_j t)\right] \cdot dt \\
&= \sum_{j=0}^{N-1} \frac{3\bar{n}_j}{2} + \bar{n}_j \delta_{kj} \\
&= \frac{3\bar{n}_{vol}}{2} + \bar{n}_k
\end{aligned} \qquad (7)
$$

Following Parseval's theorem, Eq. (7) is essentially the noise power spectrum. Therefore, removing DC component (i.e., $\bar{n}_{vol}$) during the image processing would yield the effective noise power spectrum, and thus the variance of ${\sigma_{S_k}}^2 \approx \frac{\overline{n_{vol}}}{2} + \overline{n_k}$. Here one could observe that the shot noise at each pixel on the camera depends on the photon number detected from all stacks – resulting in a cross-talk. To consider the SNR in CLAM, we arrive at:

$$
SNR_{CLAM} = \frac{\mu_{S_k}}{\sqrt{\sigma_{S_k}^2+\sigma_{D_k}^2}} = \frac{\bar{n}_k}{2\sqrt{(\bar{n}_{vol}/2+\bar{n}_k)+\sigma_D^2/N}}. \qquad (8)
$$

where σ_D is the camera noise including all readout noise sources, σ_{D_k} is the effective camera noise read out in the kth section. The normalization factor of $1/N$ arising from the Fourier transform operation that the readout noise is effectively distributed to each 'frequency bin' after Fourier transform. Compared to the laser-

scanning based LSFM, the mean signal photon number over the same volume frame period will be lowered to $\bar{n}_k/N$. Therefore, the corresponding SNR is given by

$$SNR_{LSFM} = \frac{\bar{n}_k/N}{\sqrt{\bar{n}_{vol}/N+\sigma_D^2}} = \frac{\bar{n}_k}{\sqrt{N(\bar{n}_{vol}+N\sigma_D^2)}}. \quad (9)$$

In readout-noise-limited regime, the *SNR*s are reduced to $SNR_{CLAM} = \frac{\bar{n}_k\sqrt{N}}{2\sigma_D}$ and $SNR_{LSFM} = \frac{\bar{n}_k}{N\sigma_D}$ respectively. For multiple light sheets with $N \geq 2$, CLAM has better *SNR* than LSFM. In contrast, under shot-noise-limited regime, CLAM yields better SNR when $2\left(\frac{\bar{n}_{vol}}{\bar{n}_k}+1\right) < N$ which can be approximated to $\frac{2\bar{n}_{vol}}{\bar{n}_k} < N$ as N can be as high as 30-40 as demonstrated in this work. This is the condition when the fluorescent samples are generally sparse and is applicable to many biological fluorescence imaging applications. In summary, CLAM improves the noise performance particularly when it operates in the readout-noise-limited regime. In the shot-noise-limited regime, *SNR* of CLAM scales with sparsity of the fluorescent sample with the caveat that multiplexing inherently distributes the shot noise across all the 2D stacks.

3. Key design criteria in CLAM based on OFDM

The imaging performance of CLAM for a given volume rate (f_{vol}) is thus closely linked to the characteristics of the light-sheet multiplexing. In this work, we adopted OFDM, i.e., individual light sheets are modulated by unique frequency carriers which are orthogonal to each other, because of the simple modulation implementation (simply by the spinning patterned reticle), its resilience to cross-talk among frequency carriers and its high spectral efficiency (i.e., it allows closely-spaced carriers within a given available modulation frequency bandwidth (BW))[2]. To ensure orthogonality, the volume time ($1/f_{vol}$) has to be an integer multiple of the inverse of the frequency spacing between adjacent carriers (δf), i.e., $f_{vol} = \delta f/M$, where M is an integer. To maximise the volume rate for a given δf, we chose M =1 in this work. Hence, $f_{vol} = \delta f$. Following the Nyquist sampling criterion, we set the upper limit of the modulation frequency (f_H) lower than half of the camera frame rate (f_{cam}), i.e., $f_H < f_{cam}/2$. On the other hand, the lower limit of the modulation frequency (f_L) should stay above half of the upper frequency limit, i.e., $f_L > f_H/2$, in order to minimize cross-talks from the higher harmonics of the modulation. For a given frequency bandwidth, i.e., $BW = f_H - f_L$ (set by the design of the reticle and spinning speed), the number of frequency "channels", or equivalently the number of light sheets (N) that can be allocated is $N = BW/\delta f = BW/f_{vol}$. This shows the key inter-relationships among number of required light-sheets (N), modulation frequency characteristics (BW, δf) for a given volume rate

(f_{vol}). Regarding the transverse (lateral) image resolution, CLAM is essentially diffraction-limited after deconvolution to minimize the effect of spherical aberration. Similar to all standard LSFM, the axial resolution of CLAM, on the other hand, is governed by the thickness of the light-sheet and the transfer function of the detection objective [3]. Note that this condition is generally satisfied when the the spatial separation between frequency channel, which is given by $\delta z_{ch} = \beta \delta f$ (β is the conversion factor between frequency and depth, see **Fig. 2d**), is set no greater than the thickness of the light sheet.

4. Optical layout

The overall experimental setup is detailed in **Fig. S1**. The light source of the CLAM system is a diode-pumped solid-state CW laser (wavelength, 532 nm; power, 400 mW, Shanghai Dream Laser). The collimated laser output beam, which was power-regulated by a half-wave plate in combination with a polarizing beam splitter, was expanded by a telescope T1 (f_1 = 50 mm, f_2 = 200 mm), and was then line-focused by a cylindrical lens CL1 (f_{CL} = 200 mm) into the angle-misaligned mirror pair at the device entrance O (Reflectivity R > 99.8%; Mirror separation S = 50 mm; Mirror length L = 200 mm IOS optics). Because of the minute misalignment angle α, the line-focused beam will be decomposed into a discrete set of beamlets, all of which are eventually reflected back to the entrance O, following a set of (spatially-chirped) zig-zig paths governed by their incident angles. The retro-reflected beamlets from the mirror pair can be considered as emanating from a set of virtual light sources located far away from the mirror pair. In this work, the number of retro-reflected beamlets N (and thus the virtual sources) was mainly controlled by adjusting the misaligned angle and the input light cone angle at the entrance O (controlled by a variable slit between T1 and CL1). In this work, N was chosen to range from 30 to 70. The polarizing beam splitter PBS2 and a quarter waveplate controls (maximises) the the power of the retroreflected beam. This beam was then relayed through a lens L (f = 200 mm), and a telescope T2 (f3=100mm, f4=200mm) onto the spinning reticle (i.e., light-sheet array encoder based on FDM), followed by another telescope T3 (f5= 200 mm; f6= 50 mm). The spinning reticle is positioned at the common focal plane (CFP) of both telescopes T2 and T3. We note that all the virtual sources are imaged on the different planes along the beam path, but still in the proximity of the CFP. This can be easily done by adjusting the focal length of lens L, and the mirror pair geometry [4]. This configuration is essential to ensure that all the virtual sources are imaged within the DOF of the illumination objectives. The beam was then relayed by an illumination tube lens TL1 (f=200 mm) followed by a cylindrical lens CL2 (f = 50 mm) and an illumination objective O1(20X, NA = 0.45, W.D., 8.2-6.9 mm, Nikon) to generate the light-sheet array illuminating the sample. The sample was mounted on a 3D translation stage. The detection objective O2 (10x, NA = 0.25, W.D.,

10.6 mm, Olympus) collected the multiplexed, coded fluorescent emission in the orthogonal direction, followed by a low pass filter which rejected the excitation light (a cut-off wavelength at 543 nm, Chroma). We employed an sCMOS camera (pixel size = 6.5 μm, Andor Neo 5.5) operating in the rolling shutter mode to capture the raw, multiplexed fluorescence signals through a tube lens (TL2, f = 200 mm), with a camera frame rate up to 3138 fps.

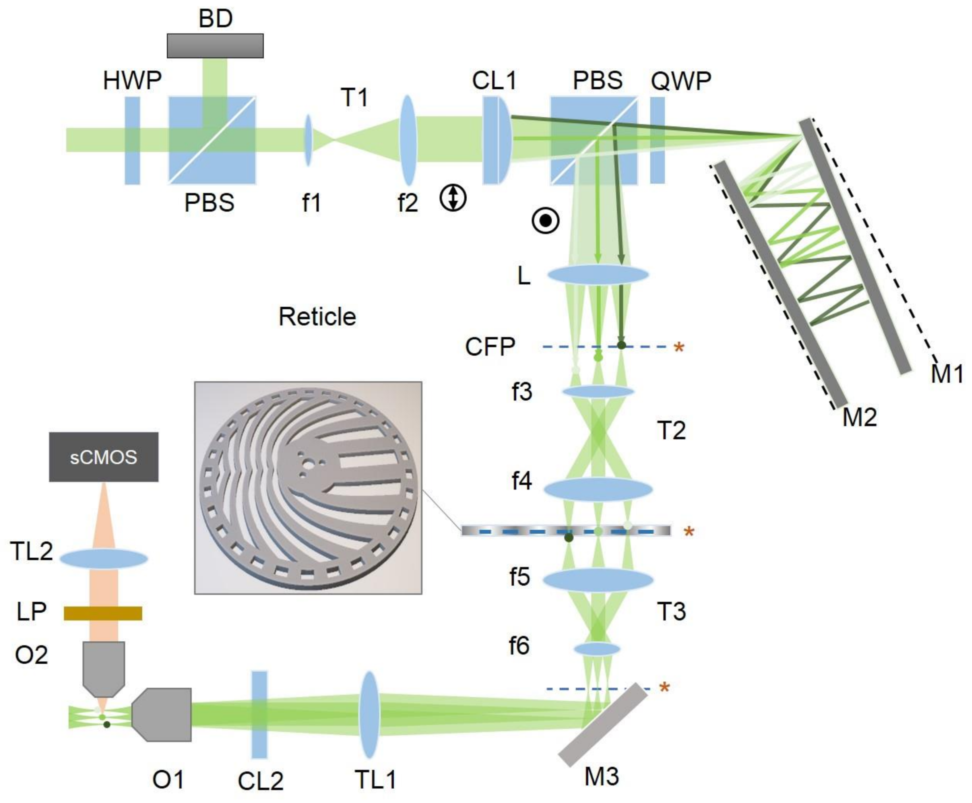

Fig. S1 System layout of CLAM. M, mirrors, f, lenses, PBS, polarizing beam splitters, $\lambda/2$ half-wave plate, $\lambda/4$, quarter-wave plate, (CL1 and CL2), cylindrical lenses, (T1, T2, and T3) telescopes. (O1 and O2), objective lenses, LP, long pass filter. Double arrow (dot) in circle shows horizontal (vertical) polarization. Positions labeled with red asterisks are conjugated planes of the virtual sources.

We adopted the OFDM concept to encode individual light sheet a unique (intensity modulation) frequency using a spinning patterned reticle. After retroreflection from the mirror pair, the beamlets are modulated by a reticle, positioned at the conjugate plane of the virtual sources. The reticle was made of a transparency film printed with a circular mask pattern (a diameter of 120 mm) (Inset in Fig. S1), defined by a transmission function, $T(r,\varphi) = \frac{1}{2} + \frac{1}{2}\mathrm{sgn}[\cos(\omega\varphi)]$, where $\omega = 2\pi r$ is the radius-dependent modulation frequency, and sgn() is the sign function. The rotating reticle (rotating speed = 120 ~ 2000 rpm) was driven by a commercial chopper motor with phase-locked rotation-speed control (MC2000B, Thorlabs). We note that

the size of the back aperture of the illumination objective lens (O1) can be varied in order to adjust the confocal parameter ("Rayleigh range") of the light-sheet array (Fig. S2).

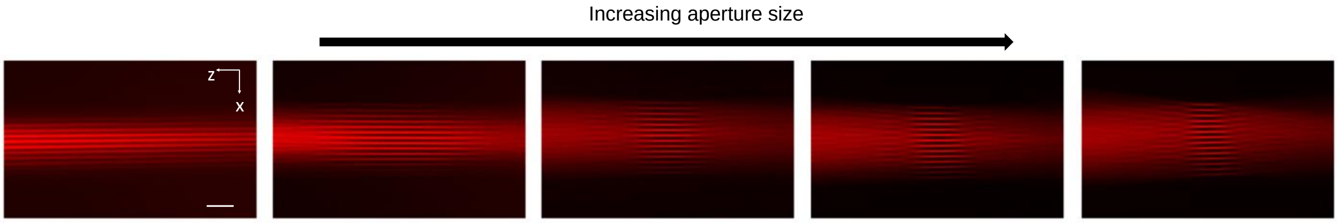

Fig. S2. Adjustment of the confocal parameter (Rayleigh range of the light sheet array) by changing the size of the back aperture of the illumination objective. Scale bar = 50 μm .

We also experimentally confirmed the concept of light-sheet array generation in CLAM using another laser source (center wavelength of 712 nm; $L_c \sim 0.1$ mm) with the mirror separation of $S \sim 20$ mm. Consistent with the performance based on the configuration shown in Fig. S1, this configuration demonstrates uniform intensity profile across the entire light sheet array (Fig. S2a-b). The thickness of individual sheets is generally preserved within the Rayleigh range defined by the illumination objective (Fig. S2a). More importantly, the light-sheet array density can be reconfigured by adjusting the mirror misalignment angle (Fig. S2b). Note that the incoherent superposition of the light sheets is clearly manifested as smooth illumination profile when the density of the light sheets is so high that individual light sheets are indistinguishable (See the rightmost profile of Fig. S2b).

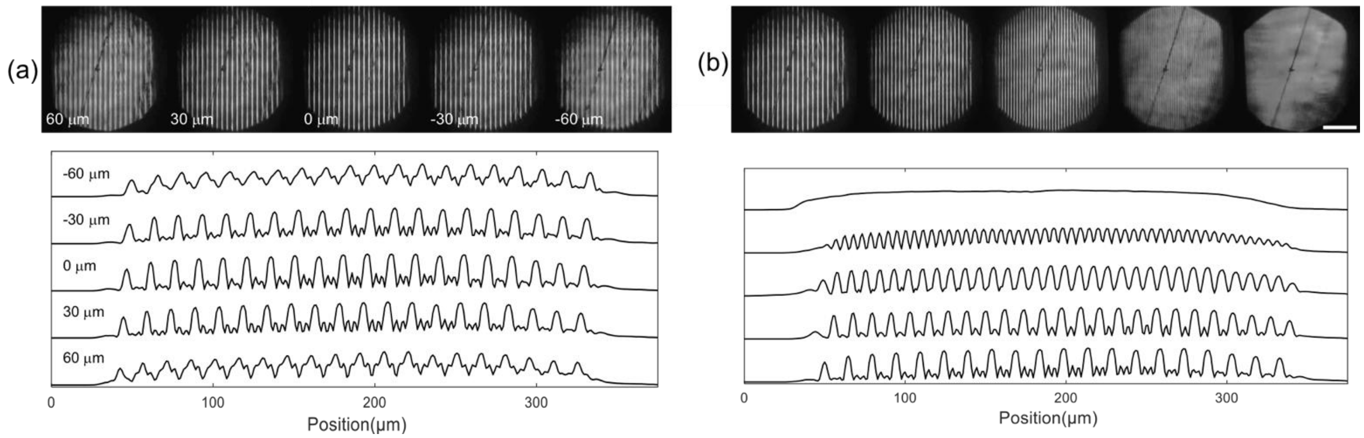

Fig. S3. Experimental evaluation of light-sheet array generation in CLAM using another laser source (center wavelength of 712 nm; $L_c \sim 0.1$ mm) with the mirror separation of $S \sim 20$ mm) (a) The light sheet array profiles at different positions along the propagation axis (x-axis) after the objective lens O1. (b) The changes in light sheet array profiles when the misaligned mirror angle is reduced (from left to right). The bottom panel shows the intensity line-profiles indicated in (a) and (b) (Yellow lines). The Scale bar represents 100 μm for (a) and (b).

5. Ray-tracing simulation of CLAM

We performed ray-tracing simulation to verify the light-sheet array generation in CLAM using Zemax. The setup configuration used in the simulation (Fig. S4) is based on the experimental setup (Fig. S1). To reduce the computational load without affecting the consistency between the simulation and experiment, the number of relay lenses was reduced in the simulation. Nevertheless, the magnification and the conjugate-plane relations are exactly the same. The purpose is to release the computational load during simulation. Inset in Fig. S4 shows the ray-tracing zoom-in view of light sheet array as well as the illumination profiles (in y-z plan) at *x*=-10 µm, *x*=0, and *x*=10µm, respectively.

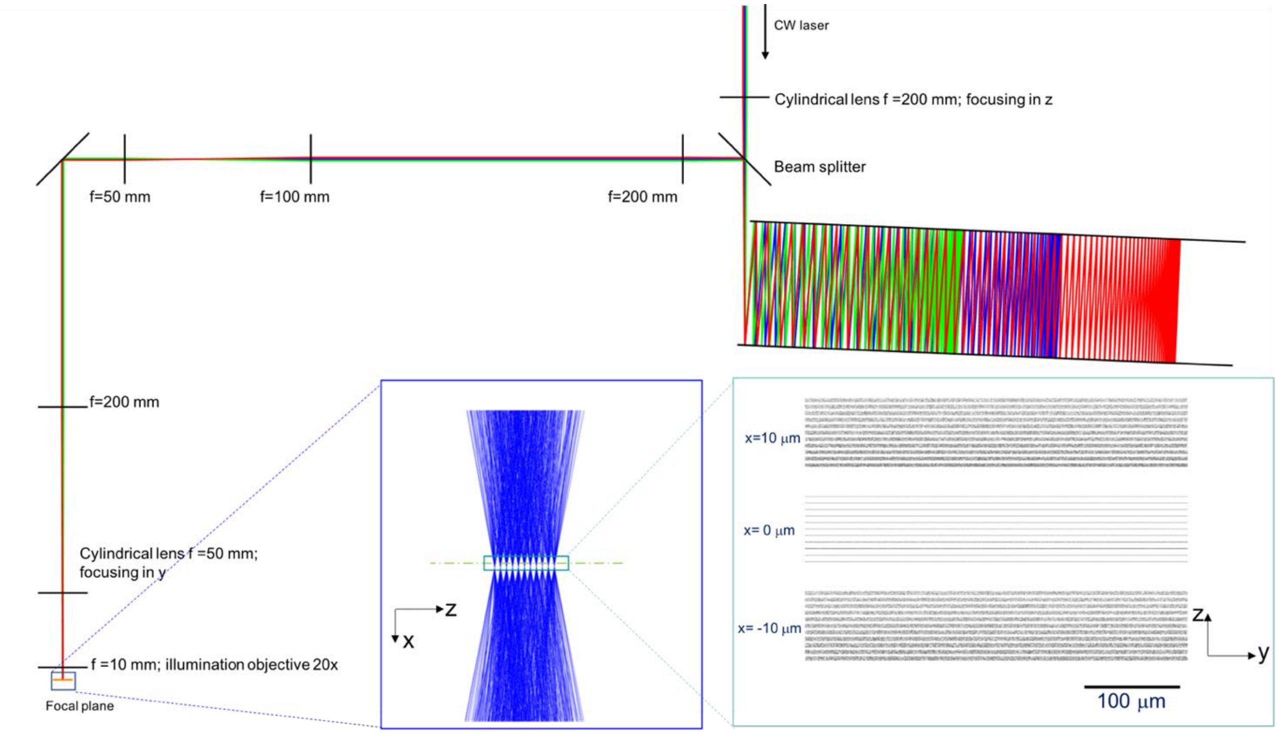

Fig. S4 Ray-tracing simulation (by Zemax) of the CLAM system based on Fig. S1. Three rays (blue, red and green), representing the paths corresponding to 3 different virtual sources, are shown for clarity. Inset shows the overall ray-tracing profile in x-z plane, and the cross-sectional profiles in y-z plane at 3 positions along x -direction (i.e., the light sheet propagation axis).

6. Characterization of the scattering medium

On average the reduced scattering coefficients of biological tissues range between $\mu_s' \sim 10 - 100\ cm^{-1}$ in the visible wavelength window[5]. We prepared the scattering sample by imbedding TiO_2 nanoparticle in the 2%

agarose gel, together with the fluorescent polystyrene beads (F8819, 1 um, Life Technologies Ltd.) as the imaging sample. The concentration of the of TiO_2 nanoparticle was chosen as 1.2 mg/mL, which corresponds to a volume fill factor of 0.00028. We further characterized the morphology of the nanoparticle and measured the size of the nanoparticles using a scanning transmission electron microscope (FEI, Tecnai G2 20). Typical images of the nanoparticle are shown in Fig. S5 at the magnification of (a) 5000x and (b) 9500x. The average size of the nanoparticle is about 160 nm. We then performed a Mie scattering calculation for nanoparticles of different diameters with the same fill factor (corresponds to a concentration of 1.2 mg/mL), the (reduced) scattering coefficients are shown in Fig. S5(c). The Mie scattering calculation[5] with the experimental volume fill factor and the particle size (160 nm diameter) suggests a reduced scattering coefficient of $\mu_s' = 22\ cm^{-1}$, similar to the reduced scattering coefficients of the biological tissue[5].

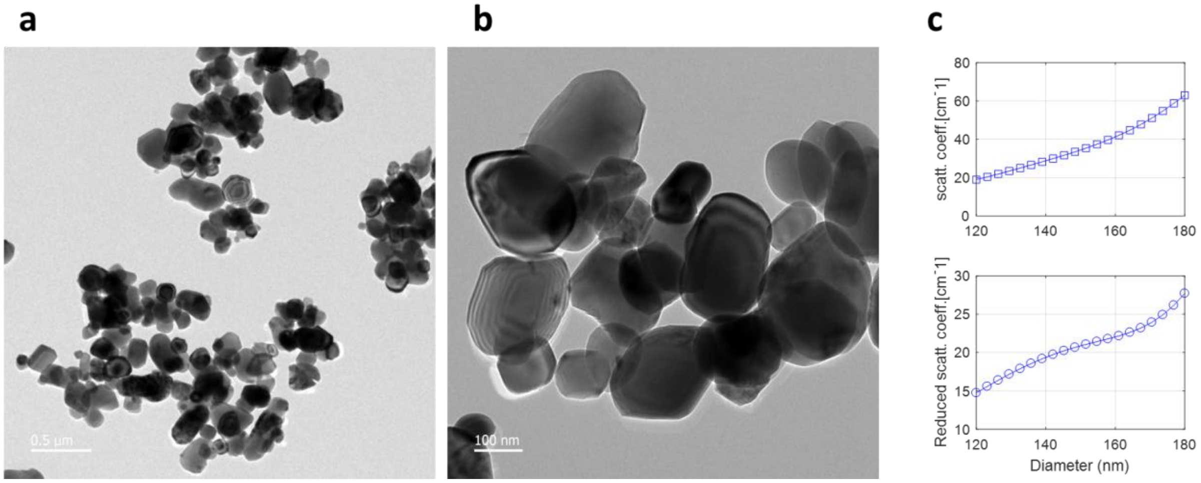

Fig. S5 The morphology of the titanium dioxide nanoparticles under (a) 5000x and (b) 9500x magnification. (c) The scattering coefficient and reduced scattering coefficient of the nanoparticles as function of the nanoparticle size with the same filling factor (concentration of 1.2 mg/mL). Scale bars (a) 500 nm, (b) 100 nm.

SUPPLEMENTARY REFERENCES

1 Driggers, R. G., Halford, C. E., Boreman, G. D., Lattman, D. & Williams, K. F. Parameters of spinning FM reticles. *Applied Optics* **30**, 887-895, doi:10.1364/AO.30.000887 (1991).

2 Nee, R. v. & Prasad, R. *OFDM for wireless multimedia communications.* (Artech House, 2000).

3 Huisken, J. & Stainier, D. Y. R. Selective plane illumination microscopy techniques in developmental biology. *Development* **136**, 1963-1975, doi:10.1242/dev.022426 (2009).

4 Wu, J.-L. *et al.* Ultrafast laser-scanning time-stretch imaging at visible wavelengths. *Light Sci Appl.* **6**, e16196, doi:10.1038/lsa.2016.196 (2017).

5 Steven, L. J. Optical properties of biological tissues: a review. *Physics in Medicine & Biology* **58**, R37 (2013).
